# The influence of eye position on the animacy impression of a cube-shaped robot in motion

**DOI:** 10.1177/20416695251323769

**Published:** 2025-03-19

**Authors:** Takahiro Kawabe, Rintaro Akiyama, Takumi Yokosaka

**Affiliations:** NTT Communication Science Laboratories, Japan

**Keywords:** animacy, eye position, gaze direction, motion, cube-type robot

## Abstract

Human observers can sometimes attribute animacy or agency to non-living objects, such as robots, perceiving them as if they were alive. In particular, the movement pattern of non-living things is a key feature for perceiving life. It is also well known that the pattern of the eyes is also an important feature for the perception of the sense of life. The present study investigated how the animacy impression of a cube-shaped robot moving along the Perlin noise trajectory could be influenced by the visual patterns of the eyes, such as eye positions and gaze directions. The eyes were presented on the top surface of the cube-shaped robot. Participants were asked to rate animacy impressions of the robot. These impressions included the impression of a live animal, having intention and moving in a self-propelled manner. These impressions were consistently higher when the eyes were presented on the side of the robot's direction of motion than when they were presented on the side orthogonal to, or opposite to, the robot's direction of motion. In general, the animacy impressions were largely comparable regardless of whether the robot's gaze direction aligned with, was orthogonal to, or opposed its motion direction. However, the impression of intention was stronger when the gaze direction at the front side of the object was consistent with the motion direction than when it was inconsistent. We discuss the evolutionary role of eye position in determining animacy impressions.

## How to cite this article

Kawabe, T., Akiyama, R., & Yokosaka, T. (2025). The influence of eye position on the animacy impression of a cube-shaped robot in motion. *i*–*Perception*, *16*(0), 1–13. https://doi.org/10.1177/20416695251323769

## Introduction

Discrimination between living and non-living things is an evolutionarily essential function for animals seeking mates, prey, or predators. Such a function likely relies on heuristic visual cues. One of the most important cues for forming animacy impressions for a non-living object is a motion trajectory. [See Parovel (2023) for a recent comprehensive review.] Human observers often perceive animacy in a robot when it moves along a 1/f trajectory, characterized by changes in the motion vector that follow a pattern in which the amplitude of each Fourier component decreases proportionally with frequency (Fukuda & Ueda, 2010). Furthermore, Fukuda and Ueda (2010) demonstrated that the impression of animacy was significantly stronger when the robot's motion followed a 1/f trajectory compared to a random trajectory, where the robot's motion vector was drawn from a Gaussian distribution. The finding may be consistent with the evidence that living animals move along the 1/f trajectory. For example, Medaka (*Oryzias latipes*) shows predatory behavior toward a moving round dot on a display next to an aquarium when the dot moves along a (1/f) trajectory (Nakayasu & Watanabe, 2014).

On the other hand, the 1/f motion trajectory is not an absolute criterion for inducing a strong impression of animacy. It has been reported that the animacy impression of a non-living object on a 1/f motion trajectory is reduced when the object's motion is synchronized with other surrounding objects (Takahashi & Watanabe, 2015). Moreover, in the smoothed version of the motion trajectory, the animacy impression is weakened (Han et al., 2023; Takahashi & Watanabe, 2015).

Not only the motion trajectory of an object but also the shape of the object is a determinant for inducing the impression of animacy in a single moving object. For example, Tremoulet and Feldman (1999) have shown that a single inanimate object can induce a higher level of animacy impression when the object changes its direction or changes its speed along its trajectory. The follow-up study (Tremoulet & Feldman, 2006) showed that the change in motion direction or speed along a trajectory served only weakly as a cue for animacy impressions when a surrounding cue promoted the attribution of the trajectory change to external events (e.g., a collision) other than the intentional trajectory change. In summary, the animacy impression of a single moving object can be determined on the basis of the motion trajectory and its interaction with surrounding information.

The animacy impression was also influenced by the interaction between the motion trajectory and the orientation of an inanimate object. Using a moving object with a bar-like shape as a stimulus (i.e., a bar-like object), Tremoulet and Feldman (1999) found that the animacy impression was higher when the motion trajectory of the bar-like object was parallel to the orientation of the object than when it was not. The preference for parallelism may be related to the bilateral symmetry of biological body shapes (Knoll & Carroll, 1999; Lemaire & Vallortigara, 2023). In indirect support of this idea, Rosa-Salva et al. (2018) showed that visually naive newborn chicks also showed a similar preference for the parallel relationship between the motion trajectory and the orientation of a moving object.

In addition to the orientation of an object, the direction of the object itself (which is different from the motion direction) is also an important feature in determining the impression of animacy and its effects on perception and cognition. In the manuscript, we distinguish between “orientation” and “direction.” Specifically, “orientation” is defined within a range of 0 to 180°, while “direction” is defined within a range of 0 to 360°. This distinction becomes clear when considering the difference between a line and an arrow. Rotating a line by 180° results in an appearance identical to its original state. In contrast, rotating an arrow by 180° produces a markedly different appearance, as the “direction” of the arrow becomes opposite to its original orientation. Thus, the direction of an object must be assessed separately from its orientation. Using stimulus displays where dart-shaped items moved randomly within a stimulus field, Gao et al. (2010) investigated factors influencing the detection of a chasing-chased dart pair. They found that detection performance decreased when the dart-shaped distractor items consistently faced a task-irrelevant static square, compared to conditions where the distractors faced in their own motion direction. This phenomenon is referred to as the “Wolfpack effect. The dart-like shape of the item likely served as a static cue to the object's direction (Jardine & Seiffert, 2011). Gao et al. (2009) demonstrated that the relationship between the direction of dart-like objects and their motion direction is crucial to the Wolfpack effect. Specifically, the effect was stronger when these directions were aligned. Importantly, the Wolfpack effect was still observed when two small dots, which can be seen as eyes, were attached to the moving disk-shaped items. Thus, the eyes may act as a cue for the static direction of the item. More recently, using dynamic cones as stimuli, Palmer et al. (2023) found that the animacy impression of the stimuli was strong when the tip of the cone tracked a task-irrelevant sphere that moved three-dimensionally around the cone. Here, the tip of the cone likely served as a directional cue to the object.

The present study investigated how factors such as eye position and gaze direction influence the animacy impression of a non-living moving object. First, we describe the reason for treating eye position as one of the experimental factors. As mentioned above, it has been shown that the Wolfpack effect was observed with the stimuli in which the two disks that could be seen as eyes were attached to the object disks (Gao et al., 2010). On the other hand, it is still unclear whether the eye position, which is likely to serve as a static directional cue, modulates the animacy impression of a single non-living moving object. In particular, it has not been clarified whether the animacy impression is degraded when the eye position as a directional cue is inconsistent with the direction of motion, that is, when the object moves toward the opposite side of the eyes. In many organisms, eyes are located on the body surface corresponding to the direction of their movement. Therefore, we hypothesized that the animacy impression would be stronger when the eyes are positioned at the front of a moving object compared to when they are positioned at the rear.

Second, we describe the reason why we chose to treat the direction of gaze as an additional experimental factor. It is known that the human eye is a strong cue for perceived animacy (Looser & Wheatley, 2010). On the other hand, it was unclear how gaze direction might influence the impression of animacy. In a study investigating the perception of the gaze direction of others, Friesen and Kingstone (1998) first showed that human observers can reflexively allocate visual attention in the direction of the gaze of others and increase performance on a letter identification task at the location where the other person looked. Yamada et al. (2008) also demonstrated that the perceived direction of apparent visual motion tends to be biased toward the spatial side indicated by another's gaze. This bias may be attributed to an attention shift driven by gaze direction. Thus, it is possible that another person's gaze direction may bias the perceived direction of a non-living object by inducing an attention shift. Moreover, the gaze direction may interact with the direction of motion of the object, leading to a modulation of the animacy impression of the object. This possibility has not been investigated empirically. We hypothesized that the gaze direction consistent with motion direction would increase the animacy impression in comparison with the gaze direction inconsistent with motion direction.

The present study focused on the different aspects of the animacy impression, referring to the previous studies that investigated the components of the impression. Jozwik et al. (2022) investigated five dimensions of animacy (“being alive,” “looking like an animal,” “having agency,” “having mobility,” and “being unpredictable”). Among them, we focused on “looking like an animal,” “having agency,” and “having mobility” in the previous study and asked our participants to rate the related three impressions such as “moving like a live animal,” “having intention,” and “moving in a self-propelled manner” in Experiment 1. Some previous studies empirically demonstrated that participants reported the different patterns of the first two impressions (“moving like a living animal” and “having an intention”) based on given visual parameters (Fukai & Terada, 2013; Takahashi & Watanabe, 2015). The third impression, “moving in a self-propelled manner,” has been studied mainly in developmental science (e.g., Luo & Baillargeon, 2004; Mascalzoni et al., 2010; Premack, 1990), but no attempt has been made to compare the three impressions simultaneously within the same experimental protocol. In Experiment 2, we asked participants to rate the consistency between gaze and motion directions to ensure that participants could explicitly use the gaze direction in the stimuli to perform the rating task.

As stimuli, we used clips recording the motion of a cube-shaped robot. We investigated the role of eye positions and gaze directions in the formation of animacy impressions in the similar situation to Fukuda and Ueda (2010), who investigated the role of the robot's motion trajectory in the formation of animacy impressions of the robot.

## Experiment 1

### Method

#### Participants

In an experimental condition where participants were asked to rate the impression of moving like a living animal, 50 females (mean age of 52.3 with *SD* of 6.78) and 50 males (mean age of 48.3 with *SD* of 8.92) participated. For reference, the mean age of citizens in Japan, where the present study was conducted, is 47.2 in 2000). In an experimental condition where participants were asked to rate the impression of having intention, 51 females (mean age 49.45 with *SD* 6.78) and 51 males (mean age 50.33 with *SD* 6.62) participated. In an experimental condition where participants were asked to rate the impression of moving in a self-propelled manner, 52 females (mean age 46.34 with *SD* 8.67) and 50 males (mean age 49.94 with *SD* 7.21) participated. Based on prior calculations using Morepower 6.0 software (Campbell & Thompson, 2012), we decided to recruit 98 participants so that the statistical analysis of the data could be performed with a power of 0.8, an effect size (*η*^2^) of 0.03, a *p*-value of .05, and an MSE of 1. Due to the late termination of the experiment, more participants actually participated in the experiment than expected, but the number of excess participants was small and was not considered to have a significant impact on the analysis. Participants were recruited online by a crowdsourcing research company in Japan and were paid for their participation. The participation fee was determined based on the company's undisclosed criteria. Only people who could participate in the experiment using their personal computer with a computer mouse were recruited. Participants were naive to the specific purpose of the experiments. Ethical approval for this study was obtained from the Ethics Committee of NTT Communication Science Laboratories) (approval number: R02-009). The experiments were conducted in accordance with the principle of the 2013 Declaration of Helsinki. Written informed consent was obtained from all participants in this study.

#### Apparatus

The experiment was conducted online, so participants used their own equipment to complete the task. However, they had to use personal computers (not smartphones or tablet-type computers) as equipment because our experimental program could only run on personal computers. Therefore, the hiring company recruited only people who could perform the task on their personal computers as participants.

#### Stimuli

**Figure 1.**
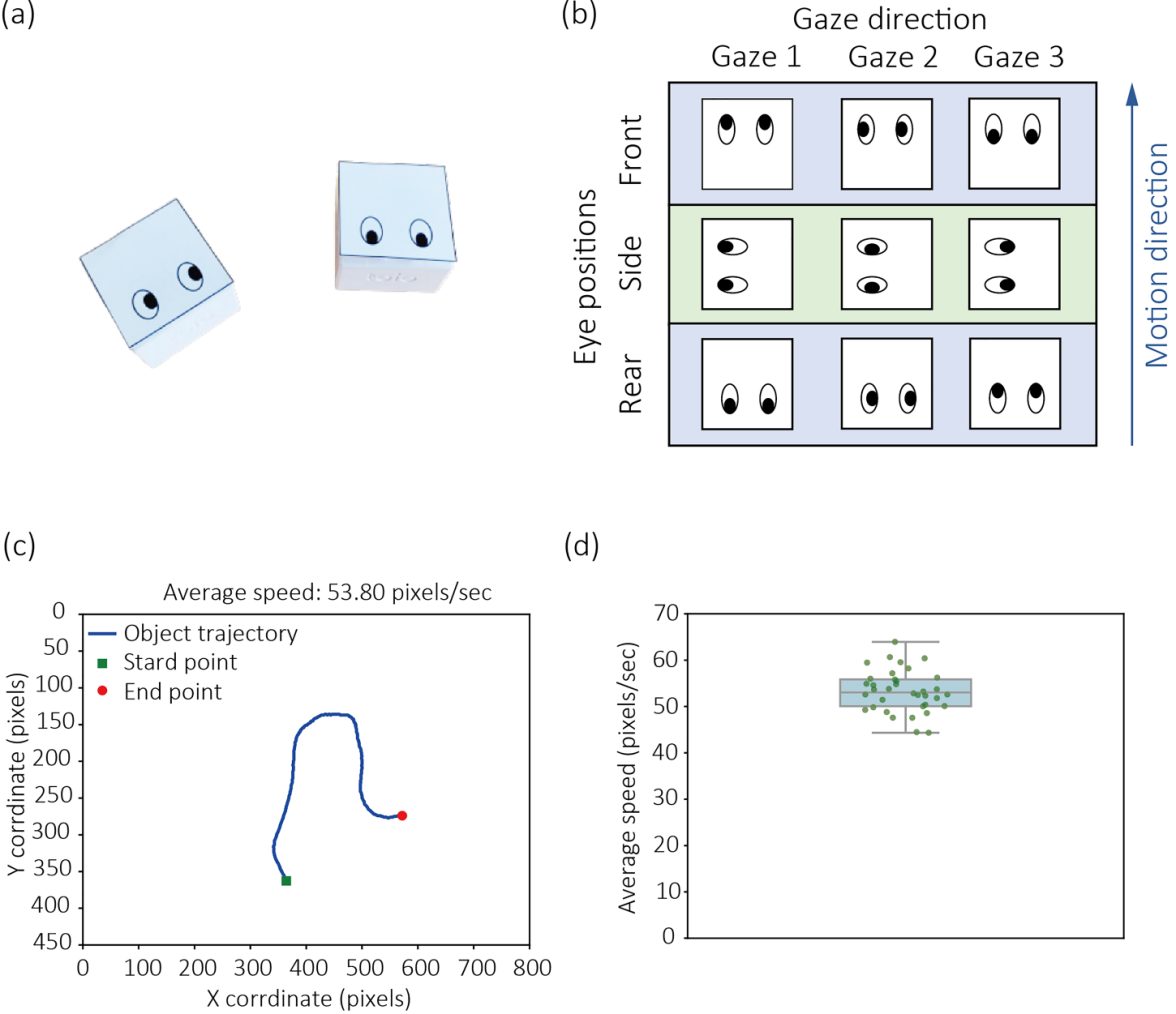
(a) The appearance of a cube-shaped robot with an eye pattern on its top surface, as shown in the stimulus clip. (b) A chart illustrating the stimulus conditions for Experiments 1 and 2, where eye positions and gaze directions were manipulated in a 3 × 3 factorial design. (c) The motion trajectory of the object in a stimulus clip. Supplemental Data 1 (https://osf.io/bz7er/) show the trajectories of an object for all stimulus clips. (d) Box and scatter plots showing the average motion speeds of the object across all stimulus clips.

We recorded videos of different scenes in which a cube-shaped robot traveled and used snippets of the scenes as stimuli (Figure 1a, Movie 1). The duration of each stimulus movie was 10 s. The spatial dimension of the top surface of the robot was 3.18 × 3.18 cm, and the vertical height of the robot was 2.56 cm. The robot had two tires at the bottom of the robot. Independent Perlin noises were used to update the rotational speed of each tire by 250 ms. The noises were generated in the range between 0 and 1, and their amplitudes were adjusted in the range between 0 and 80 and used as the tire's rotational speed (deg s^−1^). In this range of the rotational speed, the robot did not completely disappear from the video scene during the 10-s video duration. The resultant motion trajectories are provided in Supplemental Data 1 (https://osf.io/bz7er/). Although the motion trajectories were not identical across the experimental conditions tested, they were generated using the same parameters. On the top surface of the robot, we attached a paper material with a pattern printed on it that looked like eyes. As shown in Figure 1b, we controlled the position of the eye pattern in the following three conditions: In the Front condition, the eye pattern was located on the spatial side corresponding to the robot's direction of motion. In the Side condition, the eye pattern was located on the spatial side orthogonal to the robot's motion axis. In the Rear condition, the eye pattern was located on the spatial side opposite to the robot's direction of motion. Independent of the eye pattern's position, we manipulated the eye pattern's gaze direction in three ways, as shown in Figure 1b. For each combination of the position and gaze direction of the eye patterns, we tested four clips, each with a unique motion trajectory, resulting in a total of 36 clips. Each stimulus clip subtended 800 × 450 pixels. Figure 1c shows a motion trajectory of an object in a stimulus clip (see Movie 1). Figure 1d shows the distribution of average speeds among the stimulus clips. The median average speeds was 53.04 pixels/s. The spatial size of the stimulus clips was not standardized across participants, as the stimulus size varied depending on the dimensions of the monitors used by each participant.

#### Procedure

On each trial, participants viewed a stimulus clip below the instructional text. In the text, they were asked to view the clip and rate one of the following three impressions on a 7-point scale: (1) the impression of moving like a living animal, (2) the impression of having intention, and (3) the impression of moving in a self-propelled manner. Higher scores indicated stronger impressions. Non-numeric labels for positions on the scale were omitted, with only numeric score labels being displayed. Each impression was rated by an independent subset of participants. That is, each participant rated only one of the three impressions throughout the session in which they participated. They reported the rating by clicking on one of the radio buttons presented below the clip. In a 500 ms of their report, the next trial began. Each participant completed 36 trials consisting of 3 (eye positions) × 3 (gaze directions) × 4 (clip variations) in a pseudo-random order. The trials took 10–15 min to complete. All data in this study are available as Supplemental Data 2 (https://osf.io/bz7er/).

#### Statistical Analysis

Because the pattern of rating scores generally does not follow a Gaussian distribution, it is not appropriate to perform a parametric test on the raw rating data as is. Therefore, following previous studies (Elkin et al., 2021; Wobbrock et al., 2012), we used an aligned rank transformation (ART) to transform the rating scores into rank data and then used the transformed data to conduct a two-way repeated measures analysis of variance (ANOVA) with eye position (Front, Side, and Back) and gaze direction (Gaze 1, Gaze 2, and Gaze 3) as within-participant factors. Below, we report the results of the ART-ANOVA separately for each impression. The *p*-values in the post hoc tests were adjusted using the Bonferroni method.

### Results

**Figure 2.**
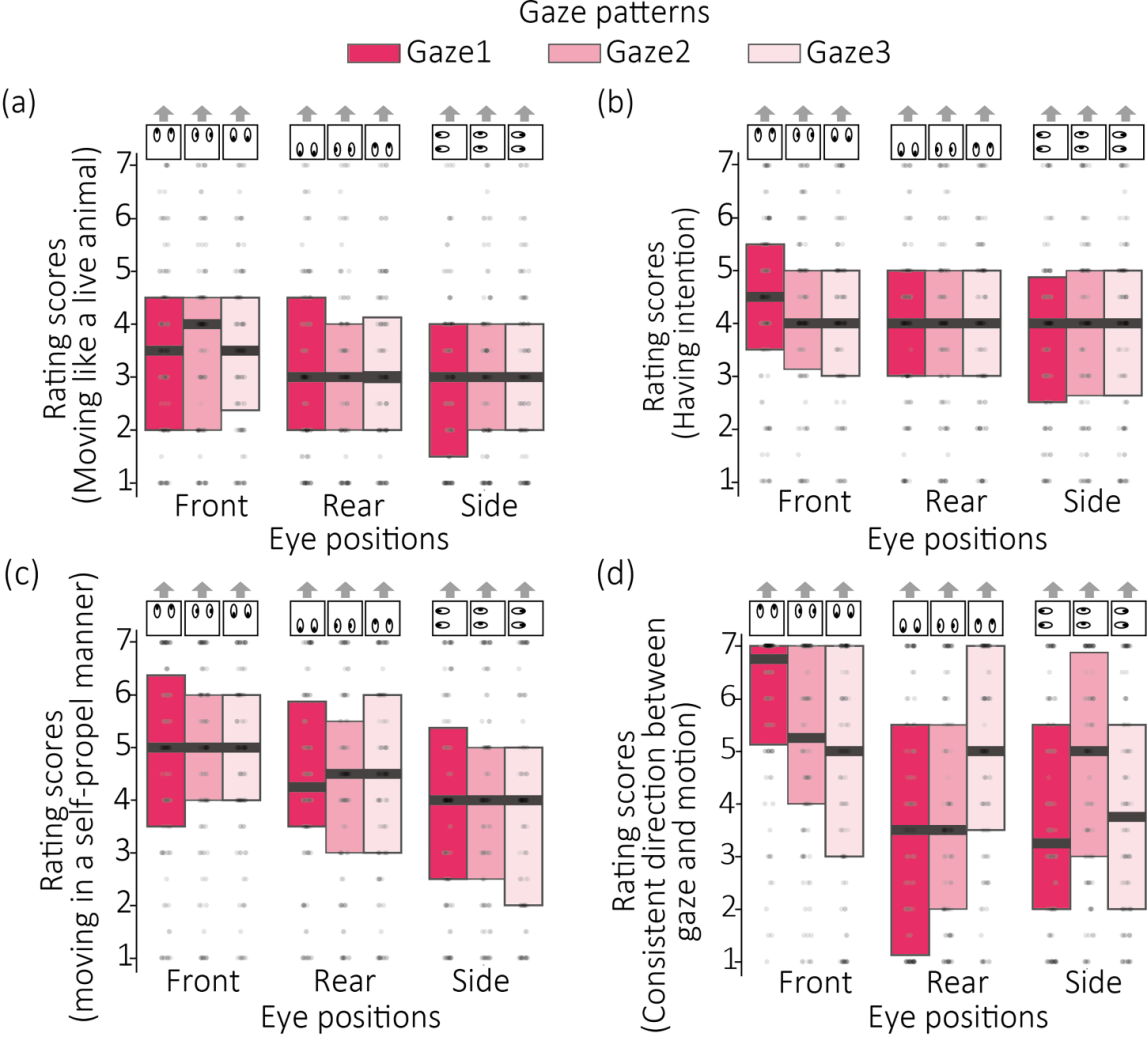
The results of (a–c) Experiment 1 and (d) Experiment 2. Rating scores for the impression of (a) moving like a live animal, (b) having intention, (c) moving in a self-propelled manner, and (d) consistent direction between gaze and motion are plotted for each condition of eye positions and gaze patterns. The lower edge of the box represents the first quartile (Q1), while the upper edge represents the third quartile (Q3). Horizontal thick lines indicate median ratings. Gray arrows above each graph indicate the motion direction of the robot.

Figures 2a–c show the box and scatter plots of the rating scores for each experimental condition. Thick horizontal lines indicate the median of the ratings.

#### Impression of Moving Like a Living Animal

The main effect of the eye position was significant [*F*(2, 198) = 24.373, *p* < .0001, *η^2^*_p_ = .170]. Multiple comparison tests with the Bonferroni correction showed that the rating scores in the Front condition was significantly higher than the scores in the Side [*t*(198) = 6.240, *p* < .0001, *d* = .321] and Rear conditions [*t*(198) = 4.269, *p* < .0001, *d* = 0.21]. The main effect of the gaze direction was not significant [*F*(2, 198) = 0.305, *p* = .731, *η^2^*_p_ = .003]. The interaction between the two factors was not significant [*F*(4, 396) = 1.145, *p* = .334, *η^2^*_p_ = .011].

#### Impression of Having an Intention

The main effect of the eye position was significant [*F*(2, 202) = 12.994, *p* < .0001, *η^2^*_p_ = .113]. The main effect of the gaze direction was not significant [*F*(2, 202) = 0.557, *p* = .573, *η^2^*_p_ = .005]. The interaction between the two factors was significant [*F*(4, 404) = 2.615, *p* = .034, *η^2^*_p_ = .025]. The simple main effect of gaze direction was significant only when the eye position was Front [*F* (2,202) = 3.4664, *p* = .0331, *η^2^*_p_ = 0.033]. Multiple comparison test of the simple main effect showed that the rating score of the Gaze 1 was significantly greater than the score of the Gaze 3 (*t*(202) = 2.631, *p* = .028, *d* = 0.152). The simple main effect of the eye position was significant when the gaze direction was Gaze 1 (*F*(2,202) = 15.718, *p* < .0001, *η^2^*_p_ = 0.134). Multiple comparison tests of the simple main effect showed that the rating score of the Front condition was significantly greater than the rating scores of the Side [*t*(202) = 5.331, *p* < .0001, *d* = 0.372] and the Rear conditions [*t*(202) = 4.169, *p* < .0001, *d* = 0.291]. The simple main effect of the eye position was significant when the gaze direction was Gaze 2 (*F*(2,202) = 4.368, *p* = .013, *η^2^*_p_ = 0.041). Multiple comparison tests of the simple main effect showed that the rating score of the Front condition was significantly greater than the rating scores of the Side condition [*t*(202) = 2.825, *p* = .0156, *d* = 0.21].

#### Impression of Moving in a Self-Propelled Manner

The main effect of the eye position was significant [*F*(2, 202) = 33.402, *p* < .0001, *η^2^*_p_ = .248]. Multiple comparison tests with the Bonferroni correction showed that the rating score in the Front condition was significantly higher than the score in the Side [*t*(202) = 8.173, *p* < .0001, *d* = .45] and Rear conditions [*t*(202) = 4.141, *p* = .0001, *d* = .231]. Moreover, the rating score in the Rear condition was significantly higher than the score in the Side condition [*t*(202) = 4.031, *p* < .0002, *d* = .225]. The main effect of the gaze direction was not significant [*F*(2, 202) = 0.0381, *p* = .962, *η^2^*_p_ < .001]. The interaction between the two factors was not significant [*F*(4, 404) = 0.432, *p* = .785, *η^2^*_p_ = .004].

### Discussion

The results showed that eye position influenced the three impressions related to animacy. Although Gao et al. (2010) elegantly demonstrated that the presence of eyes implicitly influenced animacy perception, they did not explicitly investigate how the relationship between eye position and the direction of object motion modulated the animacy impression of the object. The animacy impressions were stronger when the eyes were located on the spatial side consistent with the motion direction than when the eyes were located on the spatial side orthogonal or opposite to the direction of motion of the robot. The results suggest that eye position may serve as a static directional cue for the robot and that animacy impressions are enhanced when the static direction implied by eye position is consistent with the robot's direction of motion.

On the other hand, the difference in gaze direction was generally not a strong determinant of animacy impression ratings, even though gaze direction played an important role as a directional cue in some cognitive tasks (Friesen & Kingstone, 1998; Yamada et al., 2008). We investigated the effect of gaze direction on animacy perception because the previous studies (e.g., Gao et al., 2010) have used a pattern of eyes represented by a simple pair of disks as pupils, rather than a pattern with both sclera (white) and pupils (black) and did not examine the role of gaze directions on the animacy perception. The reason that gaze direction modulated animacy impressions less in the present study may be that the human cognitive system assumes that gaze direction changes occasionally and does not consider it as a static directional cue of the robot. The impression of having intention was influenced by the interaction between eye position and gaze direction. Thus, while gaze direction may be able to modulate animacy impressions under some conditions of eye positions, the effect of gaze direction on animacy impressions was weak, at least in our experimental setting. We will discuss this issue in the General Discussion.

One point worth addressing is the visibility of gaze direction in the stimulus clips. There was a possibility that participants did not notice the difference in gaze direction among the stimulus clips, making it difficult for them to use it as a cue to the static direction of the robot. We addressed this possibility in the next experiment.

## Experiment 2

The purpose of this experiment was to confirm whether the gaze direction in the eye pattern in the stimulus clips could be effectively used in a cognitive task other than the animacy impression rating task. In the experiment, we asked the participant to judge the consistency between the gaze direction and the motion direction of the robot. If the gaze direction was visible in the stimulus clips, the rating scores would vary depending on the interaction between eye position and gaze direction. The results would also imply that the gaze direction was visible and effectively used in calculating the consistency with the motion direction, while the cognitive system did not use the gaze direction in forming animacy impressions of the robot.

### Method

#### Participants

About 50 female (mean age of 46.14 with *SD* of 8.94) and 52 males (mean age of 48.27 with *SD* of 9.02) participated.

#### Apparatus

The apparatus was identical to that used in Experiment 1.

#### Stimuli

Stimuli were also identical to those used in Experiment 1.

#### Procedure

The procedure was also identical to Experiment 1 except for the following. In this experiment, participants were asked to rate the consistency between the direction of gaze and the direction of motion of the robot on a 7-point scale. Higher scores indicated a stronger impression of consistency.

#### Statistical Analysis

As in Experiment 1, we first transformed the rating scores using the ART, and with the transformed data, conducted a two-way repeated measures ANOVA with the eye position and the gaze direction as within-participant factors.

### Results

Figure 2d shows the box and scatter plots with median rating scores. The main effect of the eye position was significant [*F*(2, 202) = 38.78, *p* < .0001, *η^2^*_p_ = .277]. The main effect of the gaze direction was not significant [*F*(2, 202) = 1.855, *p* = .159, *η^2^*_p_ = .018]. The interaction between the two factors was significant [*F*(4, 404) = 28.882, *p* < .0001, *η^2^*_p_ = .222].

#### The Simple Main Effect of the Gaze Direction

The simple main effect of the gaze direction was significant when the position of the gaze was Front [*F*(2, 202) = 27.78, *p* < .0001, *η^2^*_p_ = .211], Rear [*F*(2, 202) = 30.625, *p* < .0001, *η^2^*_p_ = .232], and Side [*F*(2, 202) = 19.118, *p* < .0001, *η^2^*_p_ = .159]. Multiple comparison tests based on the significant simple main effect showed that in all the eye position conditions, the rating scores were significantly higher when the gaze direction was consistent with the motion direction of the robot than not (*p* < .0001).

#### The Simple Main Effect of the Eye Position

The simple main effect of the eye position was also significant when the gaze direction was Gaze 1 [*F*(2, 202) = 76.709, *p* < .0001, *η^2^*_p_ = .432], Gaze 2 [*F*(2, 202) = 16.591, *p* < .0001, *η^2^*_p_ = .141], and Gaze 3 [*F*(2, 202) = 10.011, *p* < .0001, *η^2^*_p_ = .090]. Multiple comparison test based on the significant simple main effect showed that the rating score in the Front condition was significantly higher than the scores in the Rear [*t*(202) = 10.894, *p* < .0001, *d* = 1.181] and Side [*t*(202) = 10.551, *p* < .0001, *d* = 1.143] conditions when the gaze direction was Gaze 1. Counterintuitively, in the Gaze 2 condition, the rating score in the Front condition was not significantly different from the score in the Side condition (*p* = .189) despite that in the latter condition the gaze direction was consistent with motion direction. Likewise, in the Gaze 3 condition, the rating score in the Front condition was not significantly different from the score in the Rear condition (*p* = 1.000).

### Discussion

The results showed that participants could judge the consistency between gaze direction and motion direction even with the stimulus clip used in Experiment 1, indicating that the visibility of gaze direction in the stimulus clip was sufficient for participants to perform the cognitive task for gaze direction. Thus, the weak role of gaze direction in Experiment 1 was not due to the visibility of the stimuli. Rather, it appears that the cognitive system does not use gaze direction as a cue for animacy impressions.

Interestingly, eye position also had a significant effect. Specifically, regardless of gaze direction, the eyes located in the front were judged to be consistent with the direction of motion with a subjective strength comparable to that of the eyes whose direction was consistent with the motion direction. The results suggest that both eye position and gaze direction serve as a direction that can be combined with motion in determining directional consistency. Importantly, however, eye position is likely to be a strong directional cue for the formation of animacy impressions compared to gaze direction.

## General Discussion

The present study investigated how eye patterns, such as eye position and gaze direction, could influence the formation of animacy impressions. Eye location was a primary factor in determining the impression of moving like a live animal and moving in a self-propelled manner, while it was a weak factor in influencing the impression of having an intention. Although noticeable, the gaze direction in our stimuli played only a minor role in participants’ judgments of animacy. However, it significantly influenced the impression of intention when the eyes were positioned at the front of the moving robot. Taken together, these results suggest that the eye location serves as a cue for the cognitive system to judge the static direction of an object and to determine the impression of its animacy.

Given the morphological characteristics of the eyes of primate species, it may be a reasonable finding that gaze direction had no effect on the formation of animacy impressions. Kobayashi and Kohshima (1997, 2001) reported that human eyes have a larger white sclera and a horizontally wider eye contour than other primate species. The authors discussed that humans may have evolved these eye patterns/geometries to communicate more with their gaze. On the other hand, all primate species with eyes on their faces, regardless of sclera and outline patterns different from humans, are alive, have intention (Liebal & Oña, 2018; Moore, 2016; Schel et al., 2013) and move in a self-propelled manner. Therefore, it is not the gaze direction, but the location of the eyes that may be a critical cue for animacy impressions.

Gaze direction contributed significantly, albeit weakly, to the modulation of one of the animacy impressions we investigated, namely the impression of having intention. For the other two impressions (i.e., the impression of moving like a live animal and the impression of moving in a self-propelled manner), gaze direction did not significantly modulate the ratings. We interpreted the difference in the contribution of gaze direction to the different animacy impressions as follows. In general, when an agent is performing an action, the agent monitors the progress of its own action through its eye movements (Land et al., 1999). The agent's observer uses the agent's eye movements to infer the agent's intention (Castiello, 2003; Huang et al., 2015; Hudson et al., 2009; Pierno et al., 2006). Thus, gaze direction is considered to be a cue to the action intentions of others. In general, gaze direction changes dynamically with the actions to be performed and/or the internal state of an agent's attention. In the present study, on the other hand, the gaze direction did not change but remained fixed throughout the stimulus clip. It is possible that the fixed gaze direction in the present study weakened the contribution of gaze direction to the impression of having intention. Moreover, the absence of a goal object in the stimuli may weaken the influence of gaze direction. In the Wolf Pack effect (Gao et al., 2010), the stimuli included a task-irrelevant object that could implicitly serve as an apparent goal for the object's motion. Participants might have interpreted the gaze of the moving object as being directed toward this task-irrelevant object. Therefore, it is possible that the effect of gaze direction on animacy perception is amplified when the moving object appears to gaze at a task-irrelevant object.

The present study first showed that eye position served as a static directional cue and also interacted with the motion direction. In Experiment 2, when the gaze direction coincided with the motion direction of the robot, participants consistently reported the high consistency scores. At the same time, they reported comparably high consistency scores when the eyes were located on the spatial side consistent with the robot's direction of motion, regardless of gaze direction. Thus, it is possible that the cognitive system interprets that the spatial side with the eyes corresponds in some way to the head of an object, and that the object will move in the direction of the head. On the other hand, further studies are necessary to demonstrate whether the position of the eyes is a perceptual determinant of the head/tail of the object in motion.

Finally, based on the results of this study, we would like to consider how the implementation of the robot's eyes could be improved. Based on the results of this study, we suggest that placing the robot's eyes on the side consistent with the robot's motion direction improves the robot's animacy impression. On the other hand, since the viewpoint of the video camera in our experimental setting was fixed above the desk, we had no choice but to place the eye pattern on the upper surface of the robot. This makes it difficult to directly apply the results of this study to a real robot implementation since, in a real-world setting, the user's viewpoint often changes. Regardless of the actual location of the robot's sensors, it would probably be ideal to place the eyes on the front surface of the robot. However, when the robot is viewed from behind, it is not easy for observers to determine whether the robot has eyes or not. In this scenario, the presence of eyes would not contribute to the robot's animacy. Even in this case, it might be a good idea to add another cue (like a tail) to indicate that the eyeless side is the robot's back. When we look at animals in the wild, we can tell the back of an animal by the shape of its body, the shape of its tail, and other features. We can get a lot of such information for judging body direction from the body shape design of animals, which is the result of a wonderful evolutionary process. Likewise, it may be possible to obtain useful information for designing the appearance of robots from the appearance of animals in the wild.

## Supplemental Material

**Figure:** Video 1.
